# Effects of lentiviral infection of mesenchymal stem cells on the expression of octamer transcription factor 4

**DOI:** 10.3892/mmr.2014.2505

**Published:** 2014-08-21

**Authors:** JING CHANG, LI TANG, HAN LEI, XIAO-GANG ZHANG, ZHONG ZUO, WEI HUANG, HANG FU

**Affiliations:** Department of Cardiology, The First Affiliated Hospital of Chongqing Medical University, Chongqing 400016, P.R. China

**Keywords:** green fluorescent protein, lentivirus, human umbilical cord mesenchymal stem cells, octamer transcription factor 4

## Abstract

The present study aimed to investigate the effects of lentiviral infection of human umbilical cord mesenchymal stem cells (hUCMSCs) on the expression of octamer transcription factor 4 (Oct4). hUCMSCs were infected with lentivirus carrying the green fluorescent protein gene (*GFP*) at different multiplicities of infection (MOI), and the optimal MOI was determined by flow cytometry; the proliferation of non-infected and GFP-carrying lentivirus-infected hUCMSCs was evaluated by the MTT assay; and the expression of the *Oct4* gene was measured by quantitative reverse transcription-polymerase chain reaction (qRT-PCR) and immunofluorescence staining in hUCMSCs cultured *in vitro* for eight weeks. Positive GFP staining of hUCMSCs was estimated at >75% at 48 h following infection with the GFP-carrying lentivirus (MOI = 20); no effect on hUCMSC proliferation was detected by the MTT assay following the infection; immunofluorescence analysis detected positive Oct4 expression in the cell nuclei at two and eight weeks of culture, while the relative expression of *Oct4* assessed by qRT-PCR was 0.9075±0.0124. The *GFP* gene carried by the lentivirus was successfully expressed in hUCMSCs and had no significant effect on Oct4 expression, which lays a solid foundation for future studies investigating gene functions via the use of exogenous markers.

## Introduction

Stem cell research is an emerging and actively studied topic in life sciences. Despite the pluripotency of embryonic stem cells (ESCs), their use is currently limited, largely due to ethical issues. Since 2006, transformation of fibroblasts into pluripotent cells has been successfully induced by introduction of four genes: *Oct4*, *Sox2*, *c-Myc* and *Klf4.* The resulting induced pluripotent stem (iPS) cells, have a high similarity with ESCs in morphology, proliferation, surface marker and gene expression profiles, and differentiation potential (1,2). Since then, a novel method to efficiently induce transformation of skin cells into iPS cells was also identified, involving introduction of the microRNA (mir) that is regulated by the the Oct4 and Sox2 transcription factors, miR-302 (3–5).

Human umbilical cord mesenchymal stem cells (hUCMSC), are a type of adult stem cells with multidirectional differentiation potential that can differentiate into cardiomyocytes (6), vascular endothelial cells and osteoblasts (7), etc.; this type of mesenchymal stem cells (MSCs) is widely used in research and clinical applications.

The octamer transcription factor 4 (Oct4), also known as Oct3/4 or Pou5fl (POU domain, class 5, transcription factor 1) is a homeodomain transcription factor of the POU family, and the most important transcriptional regulator of self-renewal and differentiation in ESCs. Oct4 is considered a key factor of the stem cellmultidirectional differentiation potential (8).

In this study, we infected hUCMSCs with a lentivirus that carries the green fluorescent protein gene (*GFP*) to assess its effects on Oct4 expression and explore the optimal conditions for future transfections of hUCMSCs with the GFP-conjugated exogenous mir-302.

## Materials and methods

### hUCMSC isolation and cultures

Five centimeters of the umbilical cord were collected from full-term newborns delivered by caesarean section. Informed consent of the mother, who faced no complications throughout pregnancy was obtained. The umbilical cord was immersed in a sterile culture flask containing Dulbecco’s modified Eagle’s medium (DMEM)/F12 (Gibco-BRL, Grand Island, NY, USA) and was placed on a clean bench; the arteries, veins and Wharton’s jelly were removed from the umbilical cord, followed by washing with normal saline to remove residual blood. Next, the umbilical cord was cut into 1-mm^3^ pieces and seeded in a culture flask within a small volume of DMEM/F12 medium containing 10% fetal bovine serum (FBS). The flask was placed in a 37ºC incubator for 30 min; then, part of the medium was added into wet tissue pieces, which were incubated in a humidified 37ºC, 5% CO_2_ incubator. Half of the culture medium was replaced three days later and cell growth was observed daily under an inverted microscope.

### Lentivirus packaging

A lentivirus packaging system, including pLenO-DCE-Puro, pRSV-Rev, pMDlg-pRRE and pMD2.G was purchased from (Invitrogen, Carlsbad, CA, USA). HEK-293T cells (Invitrogen) were cultured until 60–70% confluence was reached, then washed with PBS twice, isolated by treating with 0.25% trypsin containing 0.01% EDTA (Sigma, St. Louis, USA) for 6–7 minutes. Cells were counted with a hemocytometer and 2×10^6^ were seeded into 10-cm^2^ petri dishes and incubated overnight in a 37ºC incubator with 5% CO_2_. The medium was changed when the cells had reached 60–70% confluence. The 10 μl plasmid (pRSV-Rev, pMFlg-pRRE and pMD2.G; Invitrogen) and 3 μl calcium phosphate (Sigma, St. Louis, MO, USA) mixture was transferred in medium containing monolayer cells and was gently mixed; the medium was changed after 6 h of culture. The supernatant containing the virus was collected 48 h following the infection and centrifuged at 1,000 × g for 10 min at 4°C; the supernatant was passed through a 0.45-μm filter. Following a new centrifugation at 6,000 × g for 2 h at 4°C, the pellet containing the virus was dissolved in serum-free culture medium, aliquoted and stored at −70°C prior to further use.

### Lentivirus titer determination

One day before measurement, HEK-293T cells were seeded into 96-well plates with 100 μl of culture medium in each well. The lentivirus was serially diluted over the wells of the plate; 8 μf/μl polybrene (Sigma) was simulteneously added to increase the efficiency of infection. Cell growth was observed two days later and the cells were collected for subsequent titer determination.

### Measurement of infection efficiency

hUCMSCs at passage 3 were isolated as described, seeded into 24-well plates (5×10^4^ cells/well) containing DMEM/F12 medium supplemented with 10% FBS, and incubated in a 37°C incubator with 5% CO_2_. Lentivirus [multiplicity of infection (MOI)=0, 5, 10, 15, 20; triplicates for each MOI value] was added to the wells when the cells had reached 70–80% confluence. The medium was changed 24 h following the infection and the fluorescence intensity was measured after 24, 48, 72 and 96 h. Non-infected hUCMSCs were used as negative controls. The two cell groups were respectively harvested by 0.25% trypsin (Hyclone, Logan, UT, USA), digested for 2 min at room temperature and resuspended in phosphate-buffered saline (PBS) after 96 h of infection. The efficiency of infection was shown by the percentage of cells containing GFP and was calculated by the following formula: (Number of GFP positive cells/total number of cells) × 100. The cell number was determined by a BD FACSCanto II cytometer (BD Biosciences, San José, CA, USA).

### MTT assay

hUCMSCs at passage 3 were isolated as described and seeded into 96-well plates (2×10^3^ cells/well). Five wells of the non-infected and the lentivirus-infected group were used for each assay, containing PBS and an equal volume of lentivirus at a MOI of 20, respectively. At 24, 48, 72 and 96 h after the infection, 20 μl of MTT (Beyotime Institute of Biotechnology, Haimen, China) were added to each well. Following a 4-h incubation in a 37°C incubator with 5% CO_2_, the culture medium was removed and 100 μl of dimethyl sulfoxide were added to dissolve the formed crystals under low-speed vibration for 10 min. The optical density (OD) was measured at 490 nm with a microplate reader 680 (Bio-Rad, Hercules, CA, USA).

### Quantitative reverse transcription-polymerase chain reaction (qRTPCR)

Total RNA was extracted from two- and eight-week cultures of hUCMSCs with the TRIzol reagent (Invitrogen, New York, NY, USA) following the manufacturer’s instructions. The RNA was reverse transcribed into complementary DNA (cDNA) following instructions of the Takara PrimeScript TM RT-RCR kit (DRR014A; Takara Bio, Inc., Dalian, China). The forward and reverse primers were: 5′-GTGAGAG GCAACCTGGAGAAT-3; 5′-TACAGAACCACACTCGGAC CAC-3′ for the gene *Oct4* (Abcam, Cambridge, UK), and 5′-CTTTGGTATCGTGGAAGGACTC-3′; 5′-GTAGAGGCA GGGATGATGTTCT-3′ for the glyceraldehyde 3-phosphate dehydrogenase gene (*GAPDH*), respectively. The expected length of the amplified fragments was 118 and 132 bp, respectively. The qPCR reaction was performed with the SYBR Premix Ex Taq™ II kit (Bio-Rad, Hercules, CA, USA) using the following conditions: denaturation at 95°C for 30 sec, followed by 40 cycles of denaturation at 95°C for 5 sec, annealing at 55°C for 30 sec and elongation at 72°C for 30 sec. Analysis of the amplification and melting curves was performed after the reaction. The expression of *Oct4* was calculated by the Δ (ΔCt) method (9) and was expressed relative to that of *GAPDH*.

### Immunofluorescence

When cells in 24-well plates had reached 65–70% confluence, the culture medium was discarded. The plates were washed twice in PBS, fixation with 4% formaldehyde at room temperature for 20 min, three washes in PBS for 5 min, treatment with 0.2% Triton X-100 for 30 min, and three washes in PBS for 5 min. Next, blocking was performed in 1% bovine serum albumin for 1 h, and the blocking buffer was washed away prior to the incubation with the 100X-diluted rabbit anti-human/mouse Oct4 antibody (ab18976, Abcam) at 4°C in a humid box overnight; PBS instead of the primary antibody was used as the blank control. The plates were placed at 37°C for 30 min, followed by three PBS washes for 5 min, incubation with the 50x-diluted tetramethylrhodamine (TRITC)-labeled goat anti-rabbit secondary antibody IgG (Golden Bridge Biotechnology Co., Ltd., Beijing, China) in the dark at 37°C for 2 h, three PBS washes for 5 min, 4′,6-diamidino-2-phenylindole (DAPI) nuclear staining for 1 min and a final PBS wash for 5 min. Glass slides were mounted with glycerol and observed under a fluorescence microscope (Olympus, Tokyo, Japan).

### Statistical analysis

Statistical analysis was performed with the SPSS 17.0 software (IBM, Armonk, NY, USA). Quantitative data were presented as mean ± standard deviation (χ̄ ± SD), and comparisons between groups was performed with t-tests. P<0.05 was considered to indicate significant differences.

## Results

### hUCMSC growth and morphology

**Figure 1:**
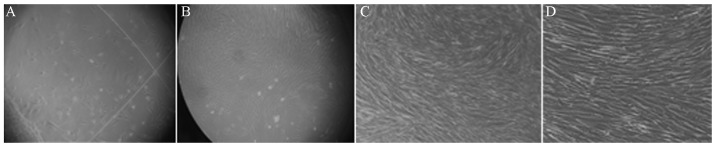
Morphology of human umbilical cord mesenchymal stem cells (hUCMSCs). (A and B) Primary culture at (A) 10 and (B) 15 days of culture; (C and D) Cells at passage 3 (C, ×100; D, ×200).

Half of the culture medium was replaced on the third day of the culture, when most of the tissue pieces had attached to the wells, and was again changed every two days afterwards. A few fusiform hUCMSCs dissociated from the tissue (Fig. 1A) after 10 days of culture, while a high number of colonies was formed after 15 days, when hUCMSCs displayed a typical fibroblast-like spindle shape (Fig. 1B). Cells were trypsinized and were passaged after they had reached 80–90% confluence. Cell morphology and properties, such as cell cycle phase (the majority were in G0/G1 phases with only a small minority in the S phase), self-renewal, pluripotency and differentiation did not significantly change after passage 10 (Fig. 1C and D).

### Analysis of hUCMSC surface markers

**Figure 2:**
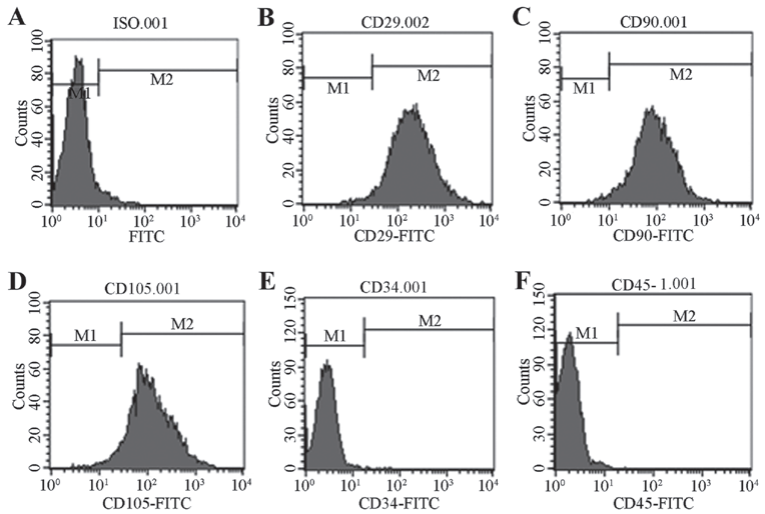
Expression of surface markers in human umbilical cord mesenchymal stem cells (hUCMSCs). hUCMSCs are positive for CD29, CD90 and CD105, and negative for CD34 and CD45.

The analysis of hUCMSCs at the third passage by flow cytometry showed that CD29, D90 and CD105 are expressed in these cells (Fig. 2A–C and E), while the cells are negative for CD34 and CD45 (Fig. 2F and G), which are the surface markers of hematopoietic stem cells.

### Lentivirus packaging and infection efficiency

**Figure 3:**
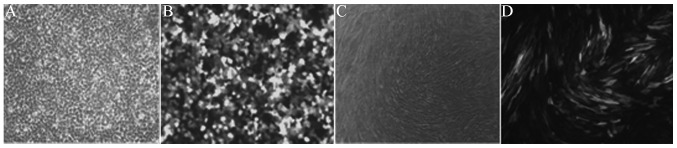
Green fluorescent protein (GFP) expression in HEK-293T cells and in human umbilical cord mesenchymal stem cells (hUCMSCs) following infection. (A and B) HEK-293T cells 48 h following the infection. (A) Light field (x100); (B) fluorescence field (x100). (C and D) hUCMSCs following the infection [multiplicity of infection (MOI) = 20, 96 h]. (C) Light field (x100); (D) fluorescence field (x100).

Bright green fluorescence was observed under the fluorescence microscope 48 h following infection of the HEK-293T cells (Fig. 3A and B), which indicated successful packaging of the active lentivirus. Hole-by-dilution was used to determine the virus titer and flow cytometry was employed to detect the percentage of GFP-positive cells. The titer of the GFP-carrying lentivirus was estimated at 2×10^8^ TU/ml, which was considered suitable for infecting hUCMSCs.

Following hUCMSC infection, all cells expressed the GFP protein as shown by green fluorescence emitted at any MOI value (except MOI = 0). The cell morphology observed under the fluorescence microscope was similar to that observed under a light microscope (Fig. 3C and D).

**Figure 4:**
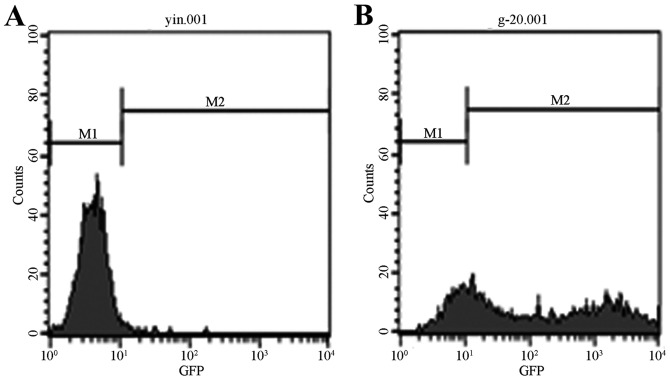
Analysis of the infection efficiency by flow cytometry. (A) Non-infected group; (B) lentivirus-infected group. The infection efficiency of the lentivirus-infected group [96 h, multiplicity of infection (MOI) = 20] is markedly higher (75.82%) compared to the non-infected group (0.75%).

The fluorescence intensity was the strongest at 96 h following the infection. With the increase of MOI and the proliferation of cells, fluorescence became stronger, reaching its highest level at MOI = 20. Flow cytometry analysis showed that the infection efficiency is 75.85% at MOI = 20 and at 96 h following the infection (Fig. 4).

### Cell proliferation

**Figure 5:**
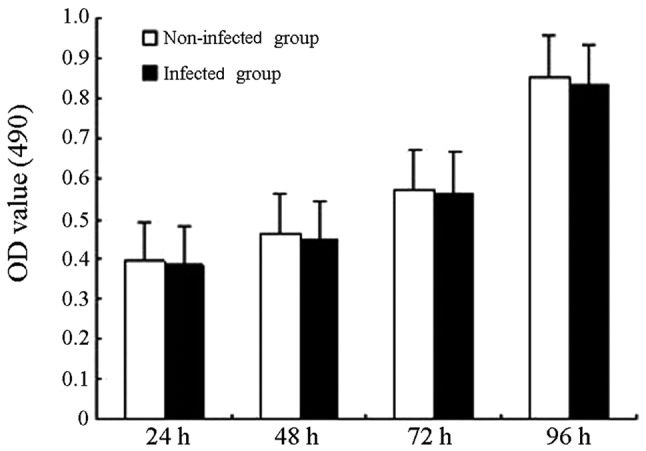
Proliferation of cells from the infected and the non-infected group, as assessed by the MTT assay. There is no statistically significant difference in the optical density (OD) values at 490 nm between the two groups.

Cell proliferation was measured in the non-infected and the lentivirus-infected group with the MTT assay (Fig. 5), and no statistically significant difference was observed between the OD values of the two groups (P>0.05), which suggested that the *GFP* gene carried by the lentivirus does not markedly affect cell proliferation under the tested conditions.

### mRNA level of Oct4

**Figure 6:**
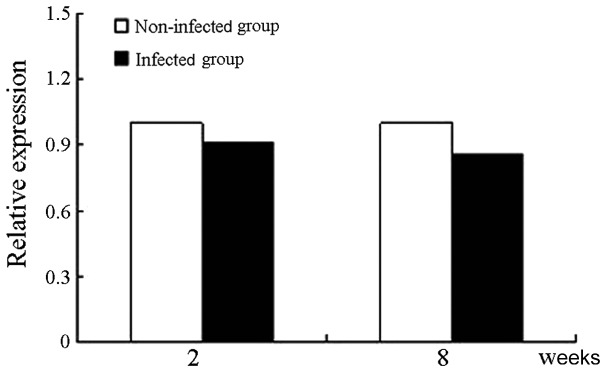
Expression of the octamer transcription factor 4 gene (*Oct4*), as assessed by quantitative reverse transcription-polymerase chain reaction (qRT-PCR). There is no statistically significant difference in the relative expression level of *Oct4* between two and eight weeks of culture.

The *Oct4* expression level was defined as 1 in the lentivirus-infected group of hUCMSCs after two weeks of culture, and thus, the level of *Oct4* was estimated at 0.9075±0.0124 after eight weeks of culture (Fig. 6). The relative expression of *Oct4* was not significantly different between cells cultured for two and eight weeks (P>0.05).

### Oct4 protein expression

**Figure 7:**
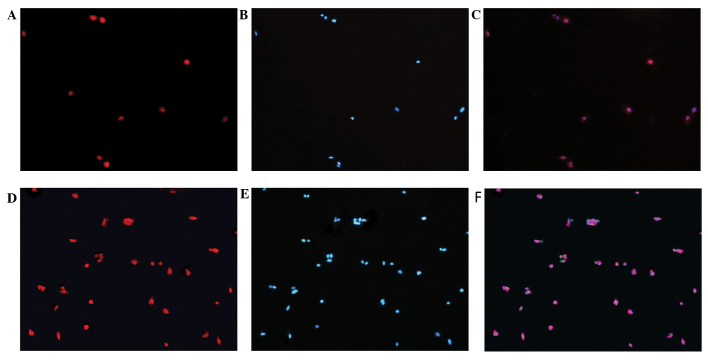
Octamer transcription factor 4 (Oct4) expression in human umbilical cord mesenchymal stem cells (hUCMSCs) observed under a fluorescence microscope (x100). (A–C) Non-infected group; (D–F) infected group. Immunofluorescence shows that the Oct4 protein is expressed in both the infected and the non-infected group. 4′,6-diamidino-2-phenylindole (DAPI) staining shows that Oct4 mainly localizes in the cell nuclei.

Immunofluorescence revealed that the Oct4 protein is expressed in hUCMSCs of both the infected and the non-infected group. There was no statistically significant difference in the levels of Oct4 between the two groups. DAPI nuclear staining experiments revealed that the Oct4 protein is mainly distributed in the cell nuclei (Fig. 7).

## Discussion

MSCs, an important member of the stem cell family, are multipotent stem cells derived from the mesoderm at the early stages of development, and show high self-renewal ability and multidirectional differentiation potential. HUCMSCs, a type of adult stem cells with multidirectional differentiation potential (10), have several advantages compared to other MSCs, including easy access and availability, high proliferation ability, low immunogenicity, and no associated ethical limitations. Moreover, hUCMSCs remain in their primitive and undifferentiated state (11–14), which renders introduction and expression of exogenous genes easy during cell proliferation; this is one reason why hUCMSCs have become the focus of numerous studies in recent years. In our experiment, we cultured umbilical cord tissue sections in order to isolate hUCMSCs.

Oct4 has been recognized as a specific marker of stem cells and a key factor for cell totipotency, which is lost during cell differentiation (15). Oct4 is not only expressed in pluripotent embryonic cells, but also in adult stem cells (16–19). Carlin *et al* (20) demonstrated that the embryonic stem cell markers Oct4, Sox2 and Nanog are expressed in hUCMSCs. Can *et al* (21) showed that the *Oct4* gene is expressed in hUCMSCs, indicating that hUCMSCs possess stem cell properties.

Lentivirus is a non-oncogenic virus; in contrast to other viral vectors, it can infect dividing and non-dividing cells, especially cells that are difficult to transfect, such as primary cells, stem cells and neurons, with an infection efficiency of almost 100%. In addition, lentiviral vectors can effectively integrate, and thus allow consistent expression of, exogenous genes into the chromosome of host cells (22,23), and have attracted increasing attention in the field of gene transfer vectors (24). Miyoshi *et al* (25) demonstrated that GFP is a marker of infection efficiency. The lentiviral vector used in our experiment bears the GFP-encoding gene, in order to allow assessment of cell infection efficiency and optimization of the infection conditions. Lentiviral vectors may integrate close to promoters and insertion mutations, which may explain why infection with the GFP-carrying lentivirus does not affect hUCMSC proliferation. The low probability of lentivirus insertion near promoters, minimizes the occurrence of insertion mutations, which may partly explain the fact that proliferation of the infected cells was not changed.

In our experiments, the *GFP* gene was successfully introduced into hUCMSCs through a lentiviral vector, which provides an ideal model for subsequent research on gene infection. Green fluorescence was observed under a fluorescence microscope and was the strongest at 96 h post-infection; the cell morphology observed under the fluorescence microscope was similar to that observed under a light microscope. Flow cytometry showed that the infection efficiency was >75% at MOI = 20. The OD values from the MTT assay did not show any significant difference between the infected and the non-infected groups, which indicates that infection with a GFP-carrying lentivirus has no effect on cell proliferation. Oct4 expression was detected by qRT-PCR and immunofluorescence. qRT-PCR revealed that the *Oct4* mRNA level is not significantly different between cells cultured for two and eight weeks, which implies that infection of hUCMSCs with the GFP-carrying lentivirus does not affect their pluripotency. Immunofluorescence further showed that the Oct4 protein is expressed in both infected and non-infected cells, with no apparent difference between the two groups, and is mainly expressed in the cell nuclei.

In conclusion, the GFP-carrying lentivirus can effectively infect hUCMSCs and has no prominent effect on cell pluripotency and proliferation. Our results lay a solid foundation for future research using exogenous gene-carrying vectors.
